# 肺癌免疫检查点抑制剂的联合治疗研究进展

**DOI:** 10.3779/j.issn.1009-3419.2020.02.05

**Published:** 2020-02-20

**Authors:** 寒菲 郭, 日兰 白, 久嵬 崔

**Affiliations:** 130021 长春，吉林大学白求恩第一医院肿瘤中心 Cancer Center, The First Bethune Hospital of Jilin University, Changchun 130021, China

**Keywords:** 肺肿瘤, 免疫检查点抑制剂, 联合治疗, Lung neoplasms, Immune checkpoint inhibitors, Combination therapy

## Abstract

检查点抑制剂（immune checkpoint inhibitors, ICIs）治疗是目前最常用的免疫治疗方案，已被批准用于黑色素瘤、肾癌、头颈癌、膀胱癌等多种肿瘤的临床治疗，在肺癌治疗中更是取得突破性进展，成为肺癌综合治疗的新支柱。但由于肿瘤的异质性及肿瘤微环境的复杂性，ICIs单药在非选择患者中有效率偏低。手术、化疗、放疗、靶向治疗等治疗手段可与免疫治疗产生协同作用，联合疗法成为目前探索的热点。本文着重于免疫检查点抑制剂联合治疗策略的研究进展，并描述了免疫疗法如何被用于早期癌症的治疗。

## Abstract (en)

Immune checkpoint inhibitors (ICIs) therapy is the most commonly used immunotherapy regimen at present. It has been approved for clinical treatment of melanoma, kidney cancer, head and neck cancer, bladder cancer and other tumors. It has made a breakthrough in the treatment of lung cancer and become a new pillar of comprehensive treatment of lung cancer. However, ICIs alone is less effective in non-selective patients, and combination therapy has become a hot topic of exploration. This article focuses on the development of combined immune checkpoint inhibitors and describes how immunotherapy can be used to treat early stage cancer.

免疫检查点作为免疫抑制性通路，对于维持自身免疫耐受和调节外周组织免疫反应的持续时间和范围至关重要。但这些通路可被肿瘤“劫持”并持续激活，抑制抗肿瘤免疫，促进肿瘤的发生^[1, 2]^。细胞毒性T淋巴细胞相关抗原4（cytotoxic T lymphocyte associated antigen-4, CTLA4）、程序性细胞死亡1（programmed death-1, PD-1）及其配体（PD-L1）是目前应用最为广泛的免疫检查点抑制剂靶点，pembrolizumab、nivolumab、atezolizumab、durvalumab和avelumab在内的PD-1/PD-L1抑制剂及CTLA-4抑制剂ipilimumab已被美国食品药品监督管理局（Food and Drug Administration, FDA）批准用于多种肿瘤的临床治疗，但由于肿瘤的异质性及肿瘤微环境的复杂性，免疫检查点抑制剂治疗的总体有效率较低^[3, 4]^。手术、化疗、放疗、靶向治疗等治疗手段可与免疫治疗产生协同作用，增强疗效，肿瘤联合治疗策略也是肿瘤治疗的未来方向。

## 1 免疫检查点抑制剂联合化疗

传统研究认为化疗直接或间接损害肿瘤特异杀伤T淋巴细胞（cytotoxic T lymphocytes, CTL），大量释放抗原引起免疫抑制等，主要对免疫系统产生抑制作用。近年来研究显示，化疗不仅能直接杀伤肿瘤，还能参与免疫系统的正向调节，改变肿瘤局部免疫微环境，如诱导肿瘤细胞发生免疫原性死亡（immunogenic cell death, ICD）^[5]^，促进肿瘤抗原及损伤相关分子模式（damage-associated molecular patterns, DAMPs）的释放，活化树突状细胞（dendritic cells, DCs），增加抗原交叉提呈; 也可以诱导局部产生CXCL10，募集T细胞至瘤床^[6]^，增强抗肿瘤特异性CTL的分化^[7]^; 化疗还可以减少免疫抑制细胞如骨髓来源的抑制性细胞（myeloid-derived suppressor cells, MDSCs）^[8]^和调节性T细胞（regulatory cells, Tregs）^[9]^。

### 1.1 临床研究进展

ICI联合化疗治疗转移性非小细胞肺癌（non-small cell lung cancer, NSCLC）的成功证明了这种双重疗法的优势^[10]^。KEYNOTE-021研究G队列中，化疗联合pembrolizumab治疗NSCLC有效率达55%，远远高于化疗组（有效率为29%），联合治疗能降低47%的疾病进展风险^[11]^。IMpower130和IMpower131研究是两项多中心、随机、开放标签的Ⅲ期临床研究，分别评估了化疗联合ICI治疗晚期非鳞NSCLC和鳞状细胞肺癌一线治疗的疗效和安全性，结果在无进展生存期（progresion-free survival, PFS）和总生存期（overall survival, OS）上都显示出了显著的临床获益，且安全性可控，进一步支持ICI联合化疗作为转移性NSCLC的一线治疗策略。基于这些研究，2018年8月，FDA批准nivolumab与培美曲塞和铂类化疗联用，在无表皮生长因子受体（epidermal growth factor receptor, *EGFR*）和间变性淋巴瘤激酶（anaplastic lymphoma kinase, *ALK*）基因变异的患者中，一线治疗转移性非鳞状NSCLC。同年10月，FDA批准nivolumab联合标准化疗一线治疗鳞状NSCLC。2019年美国临床肿瘤学会（American Society of Clinical Oncology, ASCO）会议更新Keynote-189研究结果，pembrolizumab联合化疗一线治疗晚期非鳞NSCLC，联合治疗组较化疗组中位OS（median OS, mOS）（22.0个月*vs* 10.7个月，HR=0.56，*P* < 0.000, 01）和中位PFS（median PFS, mPFS）（9.0个月*vs* 4.9个月，HR=0.48，*P* < 0.000, 01）均明显延长^[12]^，基于这一研究结果，FDA批准pembrolizumab联合培美曲塞铂类化疗用于EGFR/ALK野生型PD-L1阳性（TPS≥50%）非鳞NSCLC一线治疗。ICIs在小细胞肺癌（small cell lung cancer, SCLC）中也显示出一定的疗效，一项双盲、随机、安慰剂对照Ⅲ期研究IMpower133中，相比单纯化疗，atezolizumab联合化疗一线治疗晚期SCLC可将中位OS延长2个月，中位PFS延长0.9个月^[13]^，这在SCLC治疗史上具有里程碑意义。

### 1.2 ICIs联合化疗的相关探索

ICIs联合化疗已成为NSCLC患者标准治疗的重要组成部分，基于ICIs治疗肺癌的预测标志物的研究迅猛发展，目前，免疫组化检测PD-L1表达作为pembrolizumab治疗NSCLC被认可的伴随诊断已写入美国国家综合癌症网络（National Comprehensive Cancer Network, NCCN）指南。Pembrolizumab已获批准用于伴微卫星高度不稳定（microsatellite instability-high, MSI-H）和错配基因修复缺失（mismatch-repair deficiency, MMR）实体瘤患者治疗，作为首个FDA批准的依据生物标志物而非肿瘤类型的药物适应证，标志着肿瘤免疫治疗进入了一个全新的时代。肿瘤突变负荷（tumor mutation burden, TMB）、肿瘤浸润性CD8^+^淋巴细胞、特异基因特征^[14]^等也对ICIs治疗临床疗效有一定预测意义，有待进一步的临床数据验证。

美国东部肿瘤协作组（Eastern Cooperative Oncology Group, ECOG）体能状态（performance status, PS）评分系统在判断预后和治疗方案选择中都扮演着重要作用。ECOG PS 2分的患者占所有NSCLC患者的30%-40%，这些患者通常肿瘤负荷较大，合并其他疾病如慢性阻塞性肺疾病等，需要频繁使用皮质类固醇、抗生素等治疗，可能会影响ICI的疗效和不良反应。但ICIs在多项随机临床试验（randomized clinical trials, RCTs）中通常只入组PS评分为0分-1分的患者^[10-12]^，因此，ECOG PS 2分的患者是否可以应用ICIs是一个常见且重要的临床问题。

最近一项对18项临床试验的荟萃分析显示，接受ICIs治疗的PS 0分和1分-2分患者的OS无显著差异（*P* =0.99）^[15]^。CheckMate-171研究是一项评估nivolumab治疗包括年龄≥70岁和ECOG PS评分2分的晚期肺鳞癌患者疗效的多中心Ⅱ期研究，结果显示ECOG PS 2分的患者OS显著短于总体患者，但安全性分析无显著差异^[16]^。CheckMate-153是一项Ⅲ期/Ⅳ期临床研究，共纳入1, 375例转移性NSCLC患者接受nivolumab治疗，其中123例（8.9%）患者的PS为2分。结果显示，虽然在大多数时间点，PS 2分患者观察到肿瘤负荷显着改善，但PS 2分亚组的6个月和1年OS率低于PS 0分-1分亚组^[17]^。PePS2研究评估了pembrolizumab在ECOG PS 2分患者中的作用，在ECOG PS 2分患者人群中，总体缓解率为28.3%，中位PFS和OS分别为5.4个月和11.7个月，3级-4级不良事件为11.7%。接受多种治疗和不同PD-L1表达的患者亚组均获益^[18]^。

以上研究证明，PS 2分患者可能会对ICIs产生应答，但生存获益低于PS 0分或1分的患者。在ICIs治疗用于真实世界PS 2分的患者时，药物相关不良反应和经济负担也不容忽视。如免疫相关肺炎是一种非常严重的ICI不良反应，ICI单药治疗时肺炎的总发病率为4.1%，可严重危害已经逐渐减少的PS 2分NSCLC患者的肺储备，并且还可能造成肺炎和COPD恶化之间的鉴别诊断更加困难^[19]^。在缺乏前瞻性研究数据，特别是未与序贯治疗相比较的情况下，PS 2分患者应用ICIs联合治疗方案应谨慎。

## 2 免疫检查点抑制剂联合放疗

放疗可以诱导肿瘤细胞发生ICD，形成“肿瘤原位疫苗”，与经典的疫苗很类似，细胞外的病原相关的分子模式（pathogen associated molecule pattern, PAMP）募集单核细胞并把抗原呈递给抗原提呈细胞（antigen-presenting cells, APCs），活化的APCs进一步诱导适应性细胞免疫反应，产生放疗部位和非照射部位的缩瘤效应，后者被称为“远隔效应”。放疗还可以上调肿瘤内主要组织相容性复合体（major histocompatibility complex, MHC）和免疫共刺激分子的表达，增加促炎细胞因子如肿瘤坏死因子-α（tumor necrosis factor-α, TNF-α）、白细胞介素（interleukin, IL）-1β和黏附分子的表达，促进T细胞在肿瘤微环境中的浸润^[20]^。放疗可以重塑肿瘤微环境。激活内皮细胞，上调血管黏附分子的表达，促进异常血管正常化^[21]^; 抑制性T细胞对放疗敏感，小剂量的射线即可以清除抑制性T细胞^[22]^。研究显示，放疗24 h后发现PD-L1表达上调^[23]^，放疗导致的炎症反应可能持续数天^[24]^。因此，辐射也被认为是一种免疫佐剂，但其反应随剂量分割方案的大小而变化^[25]^，且具有时间依赖性^[26]^。

目前对于晚期NSCLC放疗联合免疫治疗的探索，放疗剂量常规分割、大分割和立体定向放射治疗（stereotactic body radiationtherapy, SBRT）均有应用，常用在免疫治疗前。PEMBRO-RT研究首次探索SBRT后pembrolizumab维持治疗在晚期肺癌的疗效，发现联合治疗较安慰剂组有效率提高一倍以上（41% *vs* 19%），且耐受性较好^[27]^。LUN 14-179研究是一项单臂Ⅱ期临床研究，结果显示晚期NSCLC患者同步放化疗后接受pembrolizumab联巩固治疗，中位至疾病转移或死亡时间（time to metastatic disease or death, TMDD）和PFS分别为22.4个月和17.0个月^[28]^。PACIFC研究共入组713例局部晚期不可切除的NSCLC患者，结果表明同步放化疗后durvalumab巩固治疗组TMDD和PFS分别为23.2个月和16.8个月，mOS较安慰剂组也显著改善（NR *vs* 28.7），且毒副反应可耐受^[29]^，基于此研究，美国FDA已经批准durvalumab用于局部晚期NSCLC患者同步放化疗后的维持治疗。在时间窗的选择上，目前研究认为放疗产生的免疫效应至1周后会大大减弱，PD-L1于放疗中或放疗后立即使用为最佳时机^[30]^。上述3项研究分别在放疗后7 d内，放疗后28 d-56 d，放疗后1 d-42 d进行ICI治疗，但选用药物不同。对于不同肿瘤类型及不同ICI药物，如何选择最佳的联合治疗时机，以最大程度增加疗效并减少不良反应目前尚无定论，仍有待更多的临床研究探索。

放疗联合ICI治疗相关性肺毒性不容忽视，PACIFC研究中免疫治疗组较安慰剂组放射性肺炎发生率增高（33.9% *vs* 24.8%），免疫治疗组3级-4级放射性肺炎发生率为4.5%，有15.4%患者因不良事件中断治疗^[29]^。LUN14-179研究中3级-4级放射性肺炎发生率为6.5%，19.6%患者因不良事件中断治疗^[28]^。因此，还需要更多的研究探索最佳的放疗剂量，联合方式及时机，标志物筛选可能获益人群也是放疗联合免疫治疗的关键。相关临床研究目前正在进行之中（表 1）：如同步放化疗后ICI维持治疗（RTOG3505研究）; 一线放化疗中同步ICI联合治疗（NICOLAS研究、DETERRED研究）; ICI诱导/巩固治疗（NCT 03102242）; ICI代替化疗与放疗联合治疗（NRG LU004 ARCHON-1研究）。

**1:** 正在进行的免疫检查点抑制剂联合放疗的临床研究 Ongoing clinical trials of Immune checkpoint inhibitors combined with radiotherapy

Clinical trials /NCT number	Phase	PD-1/PD-L1 agent	Condition or disease	Study design	Predict time of completion
RTOG 3505/NCT02768558	Phase Ⅲ	Nivolumab	Stage Ⅲ, unresectable NSCLC	4 wk-12 wk ICI as maintenance after concurrent chemoradiotherapy	January 2019
NICOLAS/NCT02434081	Phase Ⅱ	Nivolumab	Stage Ⅲa/Ⅲb, locally advanced NSCLC	Concurrent ICI to standard first-line chemotherapy and radiotherapy	August 2020
DETERRED/NCT02525757	Phase Ⅱ	Atezolizumab	Locally advanced NSCLC	Concurrent ICI to standard first-line chemotherapy and radiotherapy	January 2020
NCT 03102242	Phase Ⅱ	Atezolizumab	Stage Ⅲ, unresectable NSCLC	ICI as induction/consolidation therapy (single arm)	March 2020
NRG LU004 ARCHON-1/NCT03801902	Phase Ⅰ	Durvalumab	PD-L1 overexpression, stage Ⅱ-Ⅲ, recurrent NSCLC	ICI combined with radiotherapy	July 28, 2020
ICI: immune checkpoint inhibitor; NSCLC: non-small cell lung cancer; PD-1: programmed death-1.					

## 3 免疫检查点抑制剂联合靶向治疗

*EGFR*突变是肺癌中最常见的驱动基因，*EGFR*突变的NSCLC患者，PD-L1表达水平高低不一致，故目前研究结果仍存在争议^[31, 32]^，*EGFR*突变可导致转录因子的激活，比如STAT3、STAT1、NF-κB等分子可以穿梭进入细胞核，诱导PD-L1表达; 除了EGFR之外，其他基因TP53、KRAS、STK11等也影响PD-L1的表达，而酪氨酸激酶抑制剂（tyrosine kinase inhibitor, TKI）可增强MHC Ⅰ和Ⅱ的表达^[33]^，提高免疫提呈作用; TKI还可增强CTL介导的的抗肿瘤活性，减少T细胞凋亡^[34]^，增加IFN-γ产生; 通过促使Foxp3降解来降低肿瘤微环境中Treg细胞浸润^[35]^。鉴于TKI的多种免疫调节作用，联合免疫治疗被认为是一种很有前途的治疗方法，但目前相关研究结论不一致。针对不同的靶向药物，联合方案获得的治疗效果也有差异。

### 3.1 ICI联合EGFR-TKI治疗

多项临床试验显示，驱动基因突变的肺癌既不适合单独ICI，也不适合ICI联合TKI，原因如下：①有效率低：*EGFR/ALK*突变的晚期肺癌患者，使用PD-1抑制剂的有效率普遍低于5%，而靶向治疗的有效率高达70%以上^[31]^。②ICI联合EGFR-TKI治疗副作用明显增加，日本的一项回顾性研究纳入超过2万例EGFR突变晚期肺癌患者，所有的患者中，发生间质性肺炎或免疫性肺炎概率为4.8%，单用靶向药物或ICI治疗的概率分别是4.6%和6.4%，联合治疗为25.7%^[36]^。多项临床研究因未见明显疗效及严重副反应而提前中止（表 2）; ③爆发性进展：最近的一项临床研究分析了155例肿瘤患者接受免疫治疗的效果和基因检测结果的关系，其中2例伴有*EGFR*突变的肺腺癌患者在经过化疗和EGFR-TKI治疗之后耐药，继而使用PD-1抑制剂抗体nivolumab后现了爆发性进展，2个月之内肿瘤分别增大了53.6%和125%，增速超过治疗前的2倍以上^[37]^。

**2:** ICI联合靶向一线治疗NSCLC ICI combined with targeted drugs as first-line treatment of NSCLC

Clinical trials /NCT number	Phase	Agent	Condition or disease	ORR	Gr≥3 TRAE	Reference
TATTON	Phase Ⅰ	Osimertinib Durvalumab	EGFR (+), TKI-treatment-naive, advanced (nonresectable) NSCLC	70%	47%	[40]
CheckMate 012/NCT01454102	Phase Ⅰ	Erlotinib Nivolumab	EGFR (+), stage Ⅲb/Ⅳ, NSCLC	19%	24%	[41]
NCT02088112	Phase Ⅰ	Gefitinib Durvalumab	EGFR (+), locally advanced or metastatic NSCLC	79%	50%	[42]
NCT02013219	Phase Ⅰ	Erlotinib Atezolizumab	EGFR (+), TKI-treatment-naive, advanced (nonresectable) NSCLC	75%	39%	[43]
GEFTREM/NCT02040064	Phase Ⅰ	Gefitinib Tremelimumab	EGFR (+), have previously failed treatment with an EGFR-TKI, NSCLC	67% SD no PR/CR	54%	[44]
Checkmate 370	Phase Ⅰ/Ⅱ	Crizotinib Nivolumab	ALK rearrangement, TKI-treatment-naive, NSCLC	38%	62%	[45]
TRAE: treatment-related adverse events; ORR: overall response rate; EGFR: epidermal growth factor receptor; TKI: tyrosine kinase inhibitor.						

一些研究提示，T790M阴性者，或*KRAS*与*TP53*共突变的患者，对靶向治疗联合ICI效果较好。这可能与T790M阴性组PD-L1高表达（≥10%）和高CD8^+^ TIL浸润（≥中值）共存现象较阳性组更多（20% *vs* 4%）相关，T790M阴性组患者的FOXP3^+^ TIL计数也显著低于阳性组^[38]^。携带*KRAS*突变的患者TMB相对较高，可能与ICI治疗反应较好相关，而携带其他主要驱动基因突变（*EGFR*、*ALK*、*ROS1*等）TMB相对较低^[39]^。

综上，初治*EGFR*突变肺癌患者，靶向治疗仍是第一选择。携带驱动基因突变的患者，ICIs联合TKI疗效不确切，且安全性令人堪忧，需要进一步研究内在机制及有效的标志物，以筛选可能获益于联合治疗的人群。

### 3.2 ICI联合抗血管生成治疗

肿瘤血管的结构和功能异常导致局部缺氧和低pH值，形成抑制性肿瘤免疫微环境。缺氧可促进MDSCs的聚集，加速了肿瘤相关巨噬细胞（tumor associated macrophages, TAMs）变异和分化成免疫抑制M2表型^[46]^。缺氧还可通过上调CC-趋化因子配体的表达，间接促进Treg聚集。缺氧还可诱导肿瘤细胞上PD-L1，以及TAMs、MDSCs和Treg细胞上TIM-3和CTLA4的表达上调，并通过促进分泌VEGF，间接上调CD8^+^ T淋巴细胞上PD-1的表达，抑制免疫细胞的激活。肿瘤血管渗透性的提升和淋巴脉管的减少会造成肿瘤间质液压升高，使得免疫效应细胞更加难于进入肿瘤中心^[47]^。

抗血管生成药物通过作用于未成熟血管，促进血管正常化，可降低免疫抑制性细胞如MDSCs和Tregs活性，重塑肿瘤微环境^[48]^; 通过阻断VEGF介导的对树突细胞成熟的抑制，使得结合肿瘤抗原的T细胞更有效地启动和活化（抗原识别）。正常化肿瘤血管结构，可促进CTLs浸润进入肿瘤。但高剂量的抗血管生成药会导致过量的血管修剪，从而加剧TME的缺氧和酸中毒; 高剂量的抗VGEF药物还能增加细胞外基质的沉积，加剧局部缺氧，促进免疫抑制^[48]^。

IMpower150研究评估atezolizumab联合贝伐珠单抗/化疗治疗治疗初治Ⅳ期非鳞NSCLC的疗效和安全性，研究显示，在不同亚组患者中，atezolizumab+贝伐珠单抗+卡铂+紫杉醇相比于贝伐珠单抗+卡铂+紫杉醇，均显示出可控的安全性，以及良好的抗肿瘤活性^[49]^，为晚期非鳞NSCLC一线治疗提供了新的治疗选择。JVDF研究探索了ramucirumab联合pembrolizumab治疗晚期NSCLC等实体瘤的疗效，30%的NSCLC患者达到客观缓解，安全性可控^[50]^。ICI治疗与抗血管生成药物联用在临床研究中初显成效，有待更多的临床研究进一步验证其联合疗效。

### 3.3 ICI联合PARP抑制剂

聚腺苷酸二磷酸核糖基聚合酶（poly ADP-ribose polymerase, PARP）是一种DNA修复酶，研究显示，PARP抑制剂显著增加PD-L1表达水平^[51]^，并激活STING/TBK1/IRF3通路，增加趋化因子如CXCL10和CCL5的表达^[52]^，并诱导细胞毒性T淋巴细胞的活化和功能^[53]^。PARP抑制剂联合ICI治疗在复发卵巢癌和乳腺癌的维持治疗中以取得显著成效^[54-56]^，目前已经有3个PARP抑制剂上市：olaparib、rucaparib和niraparib。SCLC对铂类化疗敏感，且高表达PARP1^[57]^，提示DNA修复功能损伤在其中发挥重要功能。一项随机Ⅱ期临床试验显示，PARP抑制剂veliparib联合标准化疗治疗SCLC患者客观缓解率（overall response rate, ORR）达39%^[58]^。目前PARP抑制剂联合ICI治疗在SCLC中的研究仍处于早期阶段，仍需进一步探究其联合机制。除了PARP通路分子，如DDR、ATR、ATM、CHK1、MK2等的抑制剂与ICB联用的临床进展也值得关注。

## 4 ICI联合其他免疫治疗

抗肿瘤免疫反应涉及抗原识别、呈递，免疫细胞激活、浸润、杀伤肿瘤等多个环节，肿瘤细胞可通过影响免疫过程的各个环节实现免疫逃逸，因此，ICI联合其他免疫疗法也有望增强疗效。

### 4.1 ICI联合细胞因子治疗

IL、干扰素（interferon, IFN）、肿瘤坏死因子等细胞因子已被证实具有抗肿瘤作用，IL-2目前研究较深应用较广，可促进多种免疫细胞的增殖和活性，并参与抗体反应、造血和肿瘤监视。NKTR-214前体为IL-2，能直接在肿瘤微环境中激活和扩增特定的抗肿瘤T细胞和NK细胞，并增加这些免疫细胞表面PD-1的表达。PIVOT-02临床研究者中，NKTR-214联合nivolumab治疗恶性黑色素瘤ORR达53%，获得美国FDA突破性疗法认定，用以治疗晚期或转移性黑色素瘤患者。一项放疗+IL-2+检查点抑制剂的三联疗法治疗NSCLC的临床研究正在进行之中（NCT03224871）。

IL-15不激活Treg，无IL-2相关副作用，ALT-803是一种IL-15超激动剂，在一项与Nivolumab联用治疗转移性NSCLC的Ib期临床中，整体患者ORR达29%（6/21），DCR达76%（16/21），亚组分析显示，对于既往PD-1抑制剂耐药或复发的患者，ORR和DCR分别为27%（3/11）和91%（10/11）^[59]^，且相比nivolumab单药治疗没有增加不良事件的严重程度，目前Ⅱ期临床正在进行中（NCT03520686）。

### 4.2 两种免疫检查点抑制剂联合使用

CTLA-4、PD-1、淋巴细胞激活基因3（LAG-3，也被称为CD223）、Tim-3、TIGIT这5个免疫检查点目前在肿瘤免疫治疗研究领域研究较多，基于不同免疫检查点作用机制“空间”和“时间”的不同，其联合阻断可增加疗效^[60]^，但同时毒性也明显增加。

PD-1单抗和CTLA-4单抗是应用最广泛的两种免疫检查点抑制剂，其联合阻断治疗已进入Ⅲ期临床研究，CheckMate 227研究（NCT02477826）结果证实，对于晚期肿瘤突变负荷高（TMB≥10 mut/Mb）的NSCLC患者，一线nivolumab+ipilimumab的治疗疗效优于铂类为基础的双药联合化疗，ORR分别为45.3%和26.9%，并显著延长患者的mPFS（7.2个月*vs* 5.5个月，HR=0.58，*P* < 0.001）^[61]^。2019年世界肺癌大会（World Conference on Lung Cancer, WCLC）大会公布的Ⅲb期/Ⅳ期CheckMate 817研究数据显示，采用固定剂量nivolumab+低剂量ipilimumab一线治疗晚期NSLCC，总体患者mPFS为6个月，ORR为35%，中位缓解持续时间（duration of response, DOR）尚未达到。Ⅰ期CheckMate 012研究2年随访数据显示nivolumab+ipilimumab方案ORR为43%，mDOR尚未达到^[41]^。上述3项研究结果显示，nivolumab+ipilimumab双免疫联合方案治疗NSCLC持久有效，且安全性较好。

LAG-3表达于活化的T细胞、NK细胞、B细胞和浆细胞样树突状细胞，与其配体纤维介素蛋白1（fibrinogen-like protein 1, FGL1）结合后，抑制T细胞的增殖及免疫活性^[62]^。LAG-3抗体MK-4280联合pembrolizumab治疗转移性实体瘤患者的Ⅰ期/Ⅱ期临床研究（NCT02720068）正在进行之中。基于生物标志物的pembrolizumab联合MK-4280治疗晚期NSCLC的研究（KEYNOTE-495/NCT03516981）正在进行之中。此外，LAG3和PD-L1双特异性抗体FS118和MGD013，对实体瘤和血液病系统肿瘤的Ⅰ期临床研究也正在进行之中（NCT03440437, NCT03219268）。Pembrolizumab联合anti-TIGIT抗体治疗转移性实体瘤患者的Ⅰ期临床研究（CT.gov: NCT02964013）正在进行之中。

最近研究发现，在开始双联疗法之前进行TNF阻断，能防止自身免疫样的不良反应的发生，并进一步增强肿瘤特异性CD8^+^ T细胞向肿瘤微环境和引流淋巴结中的浸润，同时减缓了T细胞激活诱导的细胞死亡^[63]^。

### 4.3 ICI联合免疫激动剂

除了给T细胞“松多个刹车”，也可通过靶向刺激性检查点分子（stimulatory checkpoint molecules）（包括CD27、CD40、OX40、GITR、ICOS等）调节肿瘤免疫微环境“踩油门”，免疫激活靶点联合IO治疗相关研究目前也已进入临床试验阶段。

肿瘤内Toll样受体9（Toll like receptor 9, TLR9）能够激活幼稚pDC，生成大量的IFN-α，同时提高其他共刺激因子的表达，增强APCs向T细胞的抗原提呈，增强抗肿瘤T细胞效应^[64]^。TLR 9激动剂CMP-001联合ICI治疗肺癌的临床研究目前正在进行之中（NCT02983045, NCT03438318）。

OX40是T细胞共同刺激因子，可促进CD8^+^ T细胞的活化增殖。OX40与其激动剂型抗体相互作用也可以抑制Treg活化并介导ADCC依赖性细胞清除，下调细胞因子IL-10的表达等。但临床前研究显示，OX40抗体和PD-1抑制剂联用降低了OX40抗体的作用，先用OX40抗体，后使用PD-1抑制剂的顺序治疗显著改善了联合用药的疗效，这种现象被称为OX40+PD-1（delay）^[65]^。提示联合免疫疗法优化疗效的时机的重要性。

### 4.4 ICI联合肿瘤疫苗和溶瘤病毒

肿瘤疫苗类型较多，可以是DNA、RNA、多肽、细胞或者基因工程微生物等，能够通过激活免疫反应并形成免疫记忆，但由于疫苗无法逆转肿瘤中已存在的免疫抑制，早期的临床结果令人失望。个体化新抗原疫苗NEO-PV-01联合ICI治疗肺癌的Ⅰ期临床研究正在进行之中（NCT02897765, NCT03380871），但相关的成本和生产效率等问题需要进一步探索。

溶瘤病毒（oncolytic virus, OV）通过基因工程手段减毒增效，可将TIL招募到免疫缺陷的肿瘤中，并触发肿瘤抗原、危险信号和促炎细胞因子的释放，裂解肿瘤细胞，同时调节TME，增加ICI的疗效^[66]^。目前OV联合ICI治疗黑色素瘤^[67]^、三阴乳腺癌等实体肿瘤^[68]^已取得初步成效。溶瘤病毒（talimogene laherparepvec, T-VEC）联合pembrolizumab治疗晚期黑色素瘤的Ⅲ期研究（NCT02263508）早期结果显示，联合疗法ORR达62%，DOR达76%^[67]^。但溶瘤病毒SVV-001用于治疗SCLC的Ⅱ期临床研究因未见疗效而提前终止（KEYNOTE-034/NCT01017601），在肺癌中应用的可行性仍需进一步探索。

### 4.5 ICI联合细胞治疗

以自然杀伤细胞（natural killer cells, NK）、肿瘤浸润淋巴细胞（tumor infiltrating lymphocytes, TIL）、细胞因子诱导的杀伤细胞（cell-cytokine induced killer cell, CIK）、γδT细胞等为代表的自体免疫细胞疗法目前在肺癌治疗取得一定的进展，如CIK细胞治疗联合化疗可改善晚期NSCLC患者PFS和OS^[69]^，γδT细胞治疗晚期NSCLC的Ⅰ期临床研究表明γδT细胞免疫治疗的安全性，但有效性有待进一步验证^[70]^。一项自体自体树突状细胞（dendritic cells, DC）CIK联合PD-1抗体治疗晚期NSCLC的Ⅰ期/Ⅱ期临床研究正在进行之中（NCT03360630）。TIL疗法联合ipilimumab或nivolumab治疗实体瘤患者的Ⅰ期/Ⅱ期临床研究也在进行之中（NCT03296137）。

通过基因修饰产生的嵌合抗原受体T细胞（chimeric antigen captoror T cell, CAR-T）不依赖于MHC激活从而实现了更强的自主性, 在肺癌中也有诸多实践，但疗效不佳^[71]^。T细胞受体基因工程T细胞TCR-T（T-cell receptor engineered T cells, TCR-T）可以识别所有能被人白细胞抗原（human leukocyte antigen, HLA）呈递的抗原，包括细胞内和细胞膜上的肿瘤相关抗原，对于实体瘤更有优势，在肺癌中也有小样本临床研究证实其安全性及耐受性^[72]^，但具体效果还需进一步临床试验来证实。

## 5 ICI联合手术治疗

通常情况下，手术等组织损伤可触发炎症反应，免疫系统向Th2免疫反应变化，包括Treg活性增强和MDSC扩增。手术应激使NK细胞、T细胞功能障碍。因此，围术期又是增强免疫、减少肿瘤复发的关键期。新辅助治疗可在手术前缩小肿瘤，降低手术难度，甚至给原本无法手术的患者手术的机会; 还可清除微小转移灶，降低复发风险等。Ⅱ期临床研究CheckMate-159（NCT02259621）结果显示，NSCLC患者接受术前nivolumab新辅助免疫治疗的安全性较好，副作用较小，没有延迟手术。术中病理切片也证实肿瘤当中大量的T细胞和巨噬细胞浸润^[73]^，提示术前ICI治疗可增强抗肿瘤免疫反应。针对Ⅲa期肺癌患者的NADIM临床研究（NCT03081689）初步结果显示，术前nivolumab+卡铂+紫杉醇的方案行新辅助治疗，主要病理缓解（main pathological response, MPR）率达85.36%，病理完全缓解（pathological complete remission, pCR）率达71.4%，影像学PR率达到72%^[74]^。LCMC3是一项单臂Ⅱ期研究，评估atezolizumab新辅助治疗手术NSCLC患者的有效性和安全性，中期分析纳入了101例患者，在评估有效的77例患者中，MPR为19%，pCR率为5% ^[75]^。2019年ASCO年会公布的nivolumab或nivolumab+ipilimumab用于早期NSCLC新辅助治疗的Ⅱ期临床研究NEOSTAR（NCT03158129）结果显示，总体MPR为25%（联合组vs单药组：33% *vs* 17%），总体pCR为18%（联合组vs单药组：29% *vs* 9%）^[76]^。

综上，ICI新辅助治疗目前已取得显著成效，但仍有待大样本多中心数据进一步验证其有效性。此外，影像缓解率明显滞后于病理缓解率，给术前传统影像学评估带了挑战。NSCLC辅助免疫调节治疗的临床研究正在进行之中（如NCT02938624、NCT03217071、NCT02818920、NCT02259621）。

## 6 小结与展望

肺癌ICI免疫治疗是当前肿瘤治疗领域最具有前景的研究之一，寻找和扩大获益人群是免疫治疗时代的新挑战：①传统治疗具有免疫效应，免疫治疗与其他治疗相互作用相互影响、存在协同作用，未来有待进一步研究联合治疗的理论基础及最佳联合治疗方案; ②如何选择最佳的联合方案、最佳给药时间及方式、剂量、顺序，以最大程度发挥抗肿瘤效应、缩小不良反应; ③无论是PD-L1表达、tTMB/bTMB、MSI/MMR还是GEP，单个的标志物总有着各自的局限性。探索用于预测免疫微环境转变的生物标志物，实现个体化免疫治疗，将真正合理的联合策略和人群筛选模式推向临床试验。通过深入了解肿瘤免疫逃逸机制和肿瘤免疫微环境，将抗肿瘤疗法有机结合，为肺癌患者治疗模式带来新的改变。
